# The results of polymerase chain reaction and MALDI-TOF mass spectrometry versus phenotypic distinction between *Klebsiella pneumoniae* and *Klebsiella oxytoca*

**DOI:** 10.3389/fmicb.2025.1514643

**Published:** 2025-02-27

**Authors:** Agata Palusiak, Anna Maciejewska, Jolanta Łukasiewicz

**Affiliations:** ^1^Department of Biology of Bacteria, Faculty of Biology and Environmental Protection, University of Lodz, Łódź, Poland; ^2^Laboratory of Microbial Immunochemistry and Vaccines, Hirszfeld Institute of Immunology and Experimental Therapy, Polish Academy of Sciences, Wrocław, Poland

**Keywords:** biochemical methods, *Klebsiella oxytoca*, *Klebsiella pneumoniae*, MALDI-TOF mass spectrometry, PCR, species identification

## Abstract

**Introduction:**

*Klebsiella pneumoniae* and *K. oxytoca* are members of *Enterobacteriaceae*. They are Gram-negative, non-motile rods that are ubiquitous in the environment and part of the human intestinal microbiota. These opportunistic pathogens may cause pneumonia and urinary tract infections. *Klebsiella* species are genetically and biochemically similar; therefore, it is important to find reliable methods for their differentiation.

**Methods:**

This study presents the results of biochemical assays, PCR, and MALDI-TOF mass spectrometry (MS) performed on 35 *Klebsiella* isolates obtained from the urine of patients from central Poland.

**Results:**

Among biochemical methods, the indole test demonstrated the highest discriminatory power, whereas the determination of growth at 10°C was the least effective. For all strains biochemically identified as *K. pneumoniae*, a 108-bp amplicon was detected, indicating the presence of the *rpoB* gene in their genome. Only 12 *K. oxytoca* isolates produced a product of the *pehX* gene. All tested strains were analyzed using the MALDI-TOF Biotyper, which confirmed, with high-quality scores, their identification based on api 20E and indole tests. Strain 0.011 was identified as *Raoultella ornithinolytica*.

**Conclusion:**

MALDI-TOF MS analysis proved to be the most reliable method for identifying *K. oxytoca* and *K. pneumoniae*, with the potential for phylogroup differentiation.

## 1 Introduction

*Klebsiella oxytoca* and *K. pneumoniae* are non-motile, Gram-negative bacilli belonging to the *Enterobacteriaceae*. These species are ubiquitous in the environment where they play the role of diazotrophs (Anbuselvi et al., 2017; Brisse et al., 2006; Kovtunovych et al., 2003). Both species also colonize the skin, oropharynx, and intestines of healthy humans and animals (Yang et al., 2022). These bacteria are regarded as opportunistic pathogens that affect mainly immunocompromised patients and cause infections of the respiratory and urinary tracts (Martin and Bachman, 2018). Among clinical *Klebsiella* spp. isolates, *K. pneumoniae* predominates over *K. oxytoca* strains; however, the increasing *K. oxytoca* isolation from the samples indicates the species is a clinically significant pathogen (Gómez et al., 2021; Singh et al., 2016). *K. pneumoniae* was first isolated from a patient by Friedländer in 1982, but its present name was introduced 5 years later by Trevisan, whereas *K. oxytoca* was first described in 1886 by Flügge as *Bacillus oxytocus perniciosus,* and the current name, *K. oxytoca,* was introduced in 1956 by Lautrop (Brisse et al., 2006; Yang et al., 2022). The existence of this species was questionable for many years, and *K. oxytoca* was even regarded as a biogroup of *K. pneumonia.* Since then, the *Klebsiella* taxonomy has been significantly reorganized, e.g., some *Klebsiella* species such as *Klebsiella terrigena*, *Klebsiella planticola*, and *Klebsiella ornithinolytica* have been reassigned to the newly established genus *Raoultella* (Brisse et al., 2006).

The *K. oxytoca* group has been found as a cause of urinary tract infections or pneumonia acquired in hospitals and antibiotic-associated hemorrhagic colitis in neonates (Cuénod et al., 2021). Isolation of *K. oxytoca* from severe medical cases and its increasingly occurring multidrug resistance phenotype requires the development of a diagnostic method for quick and proper identification of the species. *Klebsiella* species are genetically similar (Bridel et al., 2021). They also possess similar phenotypic and biochemical features. Thus, the discriminatory power of 16S rRNA gene sequencing and metabolic reaction profiles may show different effectiveness depending on the species (Cuénod et al., 2021). Specific PCR conditions with species-specific primers targeting (a) gene *pehX* encoding the polygalacturonase, which cleaves a polygalacturonic chain of demethoxylated pectin, and (b) gene *rpoB* coding the beta subunit of RNA polymerase have been developed to *K. oxytoca* and *K. pneumoniae* identification. The method allowed the identification of amplicons of 343 bp for *K. oxytoca* strains and 108 bp for *K. pneumoniae* (Chander et al., 2011; Kovtunovych et al., 2003).

The results of biochemical reactions that are indicated for *K. pneumoniae* and *K. oxytoca* identification may also vary depending on the tested strains, which may result in the incorrect identification of the species (Hansen et al., 2004). Among the biochemical reactions commonly used by reference laboratories, two features can be highlighted: indole production from tryptophan and pectate degradation. These traits differentiate the *K. oxytoca* complex (positive results) not only from *K. pneumoniae* but also from the majority of *Raoultella* species. Only *Raoultella ornithinolytica* is also capable of indole production. Gas production from lactose at 44.5°C is a feature unique to *Klebsiella pneumoniae* subsp. *pneumoniae* and is not observed in the *K. oxytoca* complex or in *Raoultella* species (Hansen et al., 2004; Brisse et al., 2006). The majority of *K. oxytoca* strains are capable of growth at 10°C, whereas 93–100% of tested *Raoultella* species also demonstrate this ability. In contrast, the growth of *K. pneumoniae* at 10°C has not been commonly observed; however, this result may be method-dependent (Hansen et al., 2004).

However, it should be remembered that phylogenetic analyses have revealed that *K. pneumoniae* and *K. oxytoca* form two complexes, and each of them includes phylogenetic groups: Kp1–Kp7 and Ko1–Ko4, Ko6–Ko9, respectively (Gómez, et al., 2021; Rodrigues et al., 2019). Kp1 contains *K. pneumoniae* sensu stricto with three subspecies: *pneumoniae*, *ozaenae,* and *rhinoscleromatis*, whereas the Ko2 phylogroup includes *K. oxytoca* sensu stricto. The remaining species within each complex are as follows: *Klebsiella michiganensis* (Ko1), *K*. *spallanzanii* (Ko3), *K*. *pasteurii* (Ko4), *K*. *grimontii* (Ko6), *K*. *huaxiensis* (Ko8), *K*. *quasipneumoniae* subsp. *quasipneumoniae* (Kp2), *K*. *quasipneumoniae* subsp. *similipneumoniae* (Kp3), *K.* var*iicola* (Kp4), *K. variicola* susp. *tropicalensis* (Kp5), *K*. *quasivariicola* (Kp6), and *K*. *africanensis* (Kp7) (Bridel et al., 2021; Brisse et al., 2006; Martínez et al., 2004). Ko5 is recognized as a sub-phylogroup of Ko1, and Ko9 as a sub-phylogroup of Ko3, whereas the taxonomic status of Ko7 remains unresolved (Rodrigues et al., 2019; Yang et al., 2022). Despite the fact that each species within the *K. oxytoca* complex shows a unique phenotypic profile, such identification has usually been done only for a few strains; moreover, the results of some phenotypic tests were variable even within one phylogroup (Yang et al., 2022). Variable results were observed for *Klebsiella spallanzanii* (Ko3) and *Klebsiella grimontii* (Ko6) when dl-*α*-glycerol-phosphate utilization was tested, a characteristic that differentiates *Klebsiella huaxiensis* (Ko8 – negative result) from the *K. oxytoca* complex (Merla et al., 2019). As for the *K. pneumoniae* complex, the situation was clear only regarding the utilization of N-acetyl-neuraminic acid, a feature characteristic exclusively of Kp4, and the utilization of 4-hydroxy-l-proline, which was negative only for Kp1 within the *K. pneumoniae* complex (Rodrigues et al., 2019).

To distinguish species within the *Klebsiella oxytoca* complex, species-specific matrix-assisted laser desorption ionization-time-of-flight mass spectrometry (MALDI-TOF MS) markers were proposed by Merla et al. (2019), who identified 31 biomarkers, and by Cuénod et al. (2021), who proposed 25 ribosomal subunit proteins for the identification of different *Klebsiella* species. However, it should be noted that successful identification and reproducibility depend on the validity of the MALDI Biotyper Database. Currently, nine phylogroups of the *K. oxytoca* complex are assigned to respective *β*-lactamase gene *bla*_OXY_ variants (Cosic et al., 2021; Yang et al., 2022). Cosic et al. (2021) have recently identified species-specific gene loci and proposed their combinations (*bla*_OXY-2_, *bla*_OXY-4_, *bla*_OXY-1&5_, *bla*_OXY-6_, *leupAB*, o*rfABC*, *orfA*’) as useful in differentiating biochemically identical species: *K. oxytoca*, *K. michiganensis*, *K. pasteurii*, and *K. grimontii* (Supplementary Figure S1).

Another method applied for species identification is the Kleborate framework (Lam et al., 2021). This method allowed Stewart et al. (2022) to demonstrate species diversity among 92 isolates previously identified by a clinical laboratory as *K. oxytoca*. However, Kleborate was designed to screen genome assemblies of *Klebsiella pneumoniae* and the *Klebsiella pneumoniae* species complex (KpSC), mainly to detect and track clinically relevant AMR (acquired antimicrobial resistance) and virulence determinants (e.g., the *rmp* hypermucoidy locus and the *rmpA2* gene) from the genome database. This tool can also be used for K or O locus identification and prediction of K and O serotypes when switched to Kaptive (Wyres et al., 2016).

To gain a better understanding of the emerging pathogen *K. oxytoca* and the more commonly isolated *K. pneumoniae,* it is extremely important to use reliable methods for their identification. These studies present the results of different kinds of methods, phenotypic, molecular, and matrix-assisted laser desorption/ionization time-of-flight mass spectrometry (MALDI-TOF MS), to indicate which is the most appropriate for the species classification to the *K. pneumoniae* or *K. oxytoca* group.

## 2 Materials and methods

### 2.1 Bacterial strains

A total of 35 *Klebsiella* spp. strains (0.04, 0.05, 0.09–0.011, 0.013, 0.019, 0.021–0.025, 0.029, 0.030, 0.033, 0.034, 0.038, 0.040, 0.042, 0.045, 0.046, 0.050, 0.054, 0.055, 0.057, 0.060–0.063, 0.065, 0.067–0.069, 0.071, 0.079) were selected for the study. The species affiliation of the strains was initially estimated (by rapid identification assay) in the Synevo laboratory, from which the strains came. All 35 strains are isolated from the urine of patients from the Łódź area, Poland. The type strains of *K. oxytoca* (8724) and *K. pneumoniae* species (700603; 10031) were obtained from the American Type Culture Collection (ATCC).

### 2.2 Biochemical tests

All 35 tested strains were biochemically identified by a standardized system api 20E (BioMerieux) according to the producer’s instructions.

The indole test was performed on a bacterial culture (after incubation at 37°C for 24 h) on a medium with DL-tryptophan according to the method developed by Ewing (1986). A positive result was shown after the addition of a few drops of Kovac’s reagent and the appearance of a red-violet ring on the culture broth surface. *Klebsiella oxytoca* can produce indole from DL-tryptophan, whereas *Klebsiella pneumoniae* strains do not exhibit this ability.

Growth determination at 10°C was conducted as Naemura and Seidler described (1978) by inoculating nutrient broth (NB) with an overnight culture in NB. The tubes were incubated at 10°C for 72 h. This ability is considered characteristic of *Klebsiella oxytoca* but not of *Klebsiella pneumoniae* strains. The optical density was measured at a wavelength of 600 nm every 24 h. OD_600_ > 0.02 was defined as a positive result.

The ability of the strains to produce gas from lactose at 44.5°C was tested by using two kinds of lactose broths: EC (0.4% lactose, bile salts, casein digest, dipotassium phosphate, potassium phosphate, and sodium chloride) and 1% lactose broth (1% lactose and peptone). In each broth, a Durham tube was inserted to observe CO_2_ production. Gas production from lactose at 44.5°C is the characteristic of *Klebsiella pneumoniae* subsp. *pneumoniae* and is not observed in the *Klebsiella oxytoca* complex.

The procedures were repeated to obtain reproducible results.

### 2.3 Genetic methods

#### 2.3.1 Isolation of genomic DNA

Genomic DNA was obtained from the tested strains cultured for 18 h in L-broth by the use of a commercial Genomic Mini Kit (A&A Biotechnology) according to the manufacturer’s instructions. DNA suspended in Tris buffer (10 mM Tris, pH = 8) was stored at −20°C.

#### 2.3.2 PCR

Species (KP – *K. pneumoniae* and KO – *K. oxytoca*) specific pairs of primers targeting the *rpoB* or *pehX* gene (KP – forward [F] and reverse [R]; KO – F and R) were used for amplification:

KP (F) 5’-CAA CGG TGT GGT TAC TGA CG-3′; 5’-TCT ACG AAG TGG CCG TTT TC-3′ (R); KO (F) 5′-GAT ACG GAG TAT GCC TTT ACG GTG-3′; 5′-TAG CCT TTA TCA AGC GGA TAC TGG-3′ (R) (Chander et al., 2011). The oligonucleotides were synthesized by Bionovo. PCR amplification was performed at a final volume of 50 μL, containing 1 μL of template DNA, 1 μL of each primer, and 25 μL of HotStarTaq™ DNA polymerase Master Mix Kit (Qiagen). PCR was performed in a thermocycler (Eppendorf) according to the following conditions (Table 1).

**Table 1:** Reaction conditions for PCR.

Stage	**KP (F and R) primer**			**KO (F and R) primer**		
	Temperature	Time [min]	Numbers of cycles	Temperature	Time [min]	Numbers of cycles
Initial denaturation	95°C	15		95°C	10	
Denaturation	95°C	1	35	95°C	1	30
Annealing	55°C	1	35	55°C	1	30
Extension	72°C	2	35	72°C	2	30
Final extension	72°C	10		72°C	10	

#### 2.3.3 Electrophoresis and visualization of the PCR amplification products

PCR products were submitted to 1% agarose gel electrophoresis in TAE buffer (40 mM Tris, pH 7.6; 20 mM C_2_H_4_O_2_, 1 mM EDTA) at 70 V. The gel was stained with ethidium bromide and visualized under an ultraviolet transilluminator (GelDoc 2000). The 108-bp amplicons are expected to be generated when a *rpoB*-specific pair of primers is used. These primers have been found to be *Klebsiella pneumoniae*-specific. The 343-bp amplicons were detected when a *pehX*-specific pair of primers was applied, and these products are typical for *Klebsiella oxytoca* strains. The band position of the 108-bp and the 343-bp amplicons was established in reference to a 100–3,000-bp or 100–2000-bp DNA ladder (BLIRT DNA-GDAŃSK) used as a molecular weight standard. The procedure was repeated to obtain reproducible results and ensure good-quality product visualization.

### 2.4 Mass spectrometry analysis

Strains were also identified by using MALDI Biotyper (Bruker) at the Jagiellonian Center of Innovation (Krakow, Poland) (strains 0.010, 0.011, 0.013, 0.021, 0.050, 0.055, 0.071, 0.061, and 0.067) and Laboratory of Microbial Immunochemistry and Vaccines, Hirszfeld Institute of Immunology and Experimental Therapy, Polish Academy of Sciences (Wrocław, Poland) (all tested strains). The 18-h *Klebsiella* isolates were spotted onto a plate according to the direct transfer method with the *α*-cyano-4-hydroxycinnamic acid as a matrix (10 mg/mL in acetonitrile 50%, water 47.5%, and trifluoroacetic acid 2.5%). MALDI-TOF MS spectra were calibrated with the Bruker Bacterial Test Standard (Bruker Daltonics, Germany) with the reference masses: 3637.8, 5096.8, 5381.4, 6255.4, 7274.5, 10300.1, 13683.2, and 16952.3 Da. The MALDI-TOF mass spectra were obtained in a positive linear mode within a mass range of 2–20 kDa by using an ultrafleXtreme (Bruker, Bremen, Germany) mass spectrometer and analyzed by Biotyper software (version 3.0). The score values were interpreted as follows: ≥2.00 – high-confidence identification; 1.70–1.99 – probably genus identification. The *m/z* data obtained were analyzed in relation to the 31 markers (ions) described previously for *K. oxytoca* species complexes (Merla et al., 2019) using the Bruker Flex Analyses software (v.3.4).

## 3 Results

### 3.1 Biochemical differentiation of *Klebsiella* spp. strains

The species identification was confirmed by api 20E system. For most strains identified by the api system as *K. oxytoca*, low discrimination was obtained according to the Analytical Profile Index (Tables 2, 3). However, %id ≥80 indicated an acceptable identification. The doubtful results concerned the urease, indole, lysine, rhamnose, or Voges–Proskauer tests. As for the urease test, an extension of the incubation time to 48 h resulted in the occurrence of a positive reaction. In the case of one strain, 0.069, species identification by api 20 E was impossible (Tables 2, 3). For the reference strains, *K. oxytoca* ATCC 8724 and *K. pneumoniae* ATCC 700603, the api 20E results were accurate and did not raise any doubts.

**Table 2:** Results of biochemical methods and growth at 10°C for *Klebsiella* spp. isolates.

The strain number	Indole	Gas from lactose at 44.5°C (EC broth)	Gas from lactose at 44.5°C (broth with 1% lactose)	Growth at 10°C after 24 h (OD_600_)
0.04	**−**	**+**	−	0.0142 (−)
0.05	**−**	**+**	−	0.0106 (−)
0.09	**−**	**+**	**+**	0.3424 (+)
0.010^a^	**+**	**−**	−	0.1114 (+)
0.011	**+**	**−**	−	0.2 (+)
0.013^a^	**+**	**−**	−	0.1402 (+)
0.019^a^	**−**	**+**	−	0.02 (−)
0.021^a^	**+**	**−**	−	0.1519 (+)
0.022	**−**	**+**	**+**	0.0051 (−)
0.023^a^	**+**	**−**	−	0.0666 (+)
0.024	**−**	**+**	**+**	0.0303 (+)
0.025	**−**	**−**	**+**	0.0062 (−)
0.029	**−**	**−**	**+**	0.0116 (−)
0.030^a^	**+**	**−**	No growth	0.1181 (+)
0.033^a^	**+**	**−**	−	0.1604 (+)
0.034*	**−**	**−**	**+**	0.0088 (−)
0.038	**−**	**−**	−	0.0188 (−)
0.040	**−**	**−**	−	0.0043 (−)
0.042^a^	**+**	**−**	−	0.0987 (+)
0.045	**−**	**+**	−	0.0331 (+)
0.046	**−**	**+**	−	0.0456 (+)
0.050*	**−**	**−**	**+**	0.0272 (+)
0.054	**+**	**−**	−	0.0649 (+)
0.055^a^	**+**	**−**	−	0.0996 (+)
0.057	**−**	**+**	**+**	0.0181 (−)
0.060	**+**	**−**	−	0.1026 (+)
0.061^a^	**+**	**−**	−	0.0848 (+)
0.062	**+**	**−**	No growth	0.0301 (+)
0.063	**+**	**−**	−	0.0113 (−)
0.065	**−**	**+**	−	0.0114 (−)
0.067^a^	**+**	**−**	No growth	0.1007 (+)
0.068	**+**	**−**	No growth	0.0697 (+)
0.069 unidentified	**−**	**+**	**+**	0.02 (−)
0.071^a^	**+**	**−**	−	0.02 (−)
0.079	**+**	**−**	−	0.1056 (+)
*K*. *o.* 8724	**+**	**−**	No growth	0.0570 (+)
*K*. *p*. 700603	**−**	**+**	**+**	0.02 (−)

*K. pneumoniae* according to api E20E
*K. oxytoca* according to api 20E
Negative results of growth at 10°C
Positive results of growth at 10°C

**–** Negative result. **+** Positive result. ^a^Low discrimination by api. *Provided by Synevo Laboratory as *K. oxytoca* strain.

**Table 3:** *Klebsiella* spp. species identification—a comparison of the all methods applied.

The tested strain	Classification to *K. oxytoca* or *K. pneumoniae* complex on the basis of:					
	Biochemical reactions				Genotypic method	MALDI-TOF mass spectrometry
	Api 20E (id. quality)	Indole test	Gas from lactose at 44.5°C (48 h)	Growth at 10°C (24 h)	PCR	Biotyper (score value)
0.04	*K*. *p*. (92.4%)	*K*. *p*.	*K*. *p*.	*K*. *p*.	*K*. *p*.	*K*. *p*. (2.119)
0.05	*K*. *p*. (92.4%)	*K*. *p*.	*K*. *p*.	*K*. *p*.	*K*. *p*.	*K*. *p*. (2.263)
0.09	*K*. *p*. (97.8%)	*K*. *p*.	*K*. *p*.	*K. o.*	*K*. *p*.	*K*. *p*. (2.136)
0.010	*K*. *o.* (88.4%)	*K*. *o.*	*K*. *o.*	*K*. *o.*	*K*. *p*.	*K*. *o.*(2.29)
0.011	*K*. *o.* (96.8%)	*K*. *o.*	*K*. *o.*	*K*. *o.*	*K*. *p*.	*Raoultella ornithinolytica* (2.27)
0.013	*K*. *o.* (88.4%)	*K*. *o.*	*K*. *o.*	*K*. *o.*	*K*. *p*.	*K*. *o.* (2.29)
0.019	*K*. *p*. (85.2%)	*K*. *p*.	*K*. *p*.	*K*. *p*.	*K*. *p*.	*K*. *p*. (2.341)
0.021**	*K*. *o.*(88.4%)	*K*. *o.*	*K*. *o.*	*K*. *o.*	*K*. *p.*	*K*. *o.* (2.19)
0.022	*K*. *p*. (99.7%)	*K*. *p*.	*K*. *p*.	*K*. *p*.	*K*. *p*.	*K*. *p*. (2.112)
0.023	*K*. *o.* (87.9%)	*K*. *o.*	*K*. *o.*	*K*. *o.*	*K*. *o.*	*K*. *o.* (2.28)
0.024	*K*. *p*. (97.8%)	*K*. *p*.	*K*. *p*.	*K*. *o.*	*K*. *p*.	*K*. *p*. (2.331)
0.025	*K*. *p*. (97.8%)	*K*. *p*.	*K*. *p*. (1% lactose)	*K*. *p*.	*K*. *p*.	*K*. *p*. (2.085)
0.029	*K*. *p*. (98.4%)	*K*. *p*.	*K*. *p*. (1% lactose)	*K*. *p*.	*K*. *p*.	*K*. *p*. (2.2)
0.030	*K*. *o.* (88.4)	*K*. *o*	*K*. *o.*	*K*. *o.*	*K*. *o.*	*K*. *o.* (2.22)
0.033	*K*. *o.* (88.4)	*K*. *o.*	*K*. *o.*	*K*. *o.*	*K*. *o.*	*K*. *o.* (2.26)
0.034*	*K*. *p*. (97.8%)	*K*. *p*.	*K*. *p*. (1% lactose)	*K*. *p*.	*K*. *p*.	*K*. *p*. (2.299)
0.038	*K*. *p*. (97.8%)	*K*. *p*.	*K*. *o.*	*K*. *p.*	*K*. *p.*	*K*. *p*. (2.318)
0.040	*K*. *p.* (92.4%)	*K*. *p.*	*K*. *o.*	*K*. *p.*	*K*. *p.*	*K*. *p*. (2.352)
0.042	*K*. *o.* (88.4%)	*K*. *o.*	*K*. *o.*	*K*. *o.*	*K*. *o.*	*K*. *o.* (2.12)
0.045	*K*. *p*. (92.4%)	*K*. *p*.	*K*. *p*.	*K*. *o.*	*K*. *p.*	*K*. *p*. (2.014)
0.046	*K*. *p*. (92.4%)	*K*. *p*.	*K*. *p*.	*K*. *o.*	*K*. *p.*	*K*. *p*. (2.366)
0.050*	*K*. *p.* (97.8%)	*K*. *p.*	*K*. *p.* (1% lactose)	*K*. *o.*	*K*. *p.*	*K*. *p.* (2.37)
0.054	*K*. *o.* (97.8%)	*K*. *o.*	*K*. *o.*	*K*. *o.*	*K*. *o.*	*K*. *o.* (1.99)
0.055	*K*. *o.* (87.9%)	*K*. *o.*	*K*. *o.*	*K*. *o.*	*K*. *p.*	*K*. *o.* (2.26)
0.057	*K*. *p.* (97.8)	*K*. *p.*	*K*. *p.*	*K*. *p.*	*K*. *p.*	*K*. *p*. (2.331)
0.060	*K*. *o.* (96.8%)	*K*. *o.*	*K*. *o.*	*K*. *o.*	*K*. *o.*	*K*. *o.* (2.37)
0.061	*K*. *o.* (88.4%)	*K*. *o.*	*K*. *o.*	*K*. *o.*	*K*.*o./K.p.*	*K*. *o.* (2.15)
0.062	*K*. *o.* (96.8%)	*K*. *o.*	*K*. *o.*	*K*. *o.*	*K*. *o.*	*K*. *o*. (2.31)
0.063	*K*. *o.* (96.8%)	*K*. *o.*	*K*. *o.*	*K*. *p*.	*K*. *o.*	*K*. *o.* (2.27)
0.065	*K*. *p.* (99.9%)	*K*. *p.*	*K*. *p.*	*K*. *p.*	*K*. *p.*	*K*. *p*. (2.001)
0.067	*K*. *o.* (88.4%)	*K*. *o.*	*K*. *o.*	*K*. *o.*	*K*.*o./K.p.*	*K*. *o.* (2.12)
0.068	*K*. *o.* (96.8%)	*K*. *o.*	*K*. *o.*	*K*. *o.*	*K*. *o.*	*K*. *o.* (2.238)
0.069**	Unidentified	*K*. *p.*	*K*. *p.*	*K*. *p.*	*K*. *p.*	*K*. *p*. (2.162)
0.071	*K*. *o.* (88.4%)	*K*. *o.*	*K*. *o.*	*K*. *p.*	*K*. *p.*	*K*. *o.* (2.11)
0.079	*K*. *o.* (98.5%)	*K*. *o.*	*K*. *o.*	*K*. *o.*	*K*. *o.*	*K*. *o.* (2.25)

*provided from Synevo laboratory as *K. oxytoca* strain, **provided from Synevo laboratory as *K. pneumoniae* strain. The data marked in green means that the species identification was inconsistent with the other methods. api 20E - the %id ≥80 indicated an acceptable identification; %id ≥90 means good quality of identification. Score values interpretation: ≥2.00 – high-confidence identification; 1,70 – 1,99 – probably genus identification. (1% lactose) – refers to the strains, which produced gas from lactose in 1% lactose broth (1% lactose, peptone). The remaining results concern the strains producing gas from lactose at 44.5 °C in EC broth (0.4% lactose, bile salts, casein digest, dipotassium phosphate, potassium phosphate, sodium chloride).

For further biochemical identification of the tested strains, three biochemical tests (indole test, growth at 10°C, and gas from lactose at 44.5°C) were chosen from the test panel for the differentiation of *Klebsiella* species. Among the strains identified as *K. oxytoca* by Synevo, only one, 0.050, did not produce indole from tryptophan, which is not a feature typical for this species (Table 2). The reference strain *K. oxytoca* ATCC 8724 produced indole, which was indicated by the presence of a red-violet ring on the surface of the culture broth, whereas *K. pneumoniae* ATCC 700603 gave a negative result in the indole assay.

As can be seen in Table 2, the ability to produce gas from lactose at 44.5°C appeared to be medium-dependent. More strains classified as *K. pneumoniae* (62.5%) produced CO_2_ when grown in EC broth than when cultured in 1% lactose broth (50%) (Table 2). None of the *K. oxytoca* strains tested in the studies occurred to be gas producer, which is consistent with the results for the *K. oxytoca* reference strain, 8724. *K. pneumoniae* ATCC 700603 produced gas in both types of media (Table 2).

When the growth response in nutrient broth at 10°C was tested after 24 h of incubation, most *K. oxytoca* strains (16 tested strains and *K. oxytoca* ATCC 8724) and 5 *K. pneumoniae* isolates were able to grow–OD values of the cultures were higher than the OD of 0.02 (Table 2). *K. pneumoniae* ATCC 700603 strain was unable to grow at 10°C. After 48 h of incubation, growth was observed for most of the tested strains except *K. oxytoca* (0.022 and 0.063). When the time of incubation was extended to 72 h, only one strain, *K. oxytoca* 0.063, was unable to grow.

### 3.2 Differentiation of *Klebsiella* spp. strains by polymerase chain reaction

**Figure 1:**
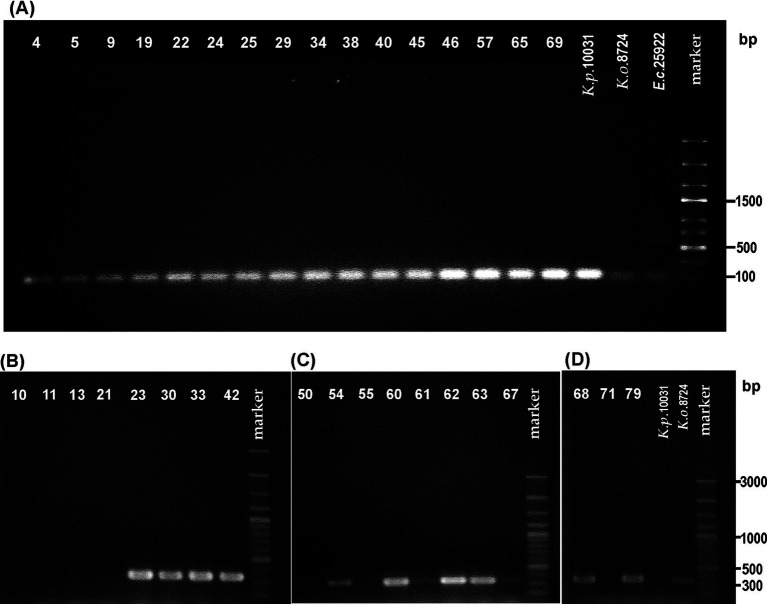
Visualization of electrophoretically separated PCR products of: **(A)** *rpoB* gene (expected size: 108 bp) for the strains biochemically identified as *Klebsiella pneumoniae*, **(B–D)** *pehX* gene (expected size: 343 bp) for the strains biochemically identified as *Klebsiella oxytoca.* The strain numbers are simplified (e.g., 0.010–10) to make the figure clearer. The band position of the amplicon was established in reference to 100–3,000-bp DNA or 100–2000-bp ladders used as a molecular weight standard. The results of the PCR control targeting the *pehX* and *rpoB* genes are presented in Supplementary Figure S1.

All strains identified by api 20E as *K. pneumoniae* and the species type strain were screened for the presence of the *rpoB* gene with primer pairs (KP(F) and KP(R)). The 108-bp fragment regarded as characteristic of the *K. pneumoniae* species was obtained from the DNA of all tested strains, which stays in agreement with biochemical identification [api 20E, indole assay, and gas production from lactose] (Figure 1A and Table 2). For *K. oxytoca* ATCC 8724 and *E. coli* ATCC 25966 (negative controls), slightly visible bands were obtained in gel (Figure 1A). For one strain, 0.034, identified by the laboratory as *K. oxytoca*, the results of both molecular and biochemical studies (including api 20E) appeared to be typical for *K. pneumoniae*.

**Figure 2:**
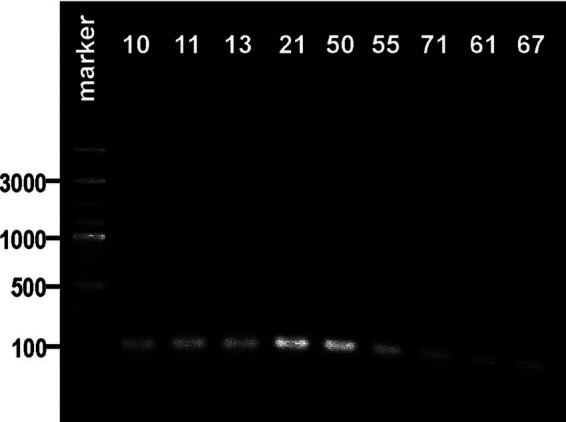
Visualization of electrophoretic separated PCR products of the *rpoB* gene (expected size: 108 bp) for the strains biochemically identified as *Klebsiella oxytoca* in agarose gel. The strain numbers are simplified (e.g., 0.010–10) to make the figure clearer. The band position of the amplicon was established in reference to a 100–3,000-bp DNA ladder used as a molecular weight standard. The results of the PCR control targeting the *pehX* and *rpoB* genes are presented in Supplementary Figure S1.

Subsequently, 18 strains biochemically classified as *K. oxytoca* (also the ones for which the api results were doubtful) and strain 0.050 (identified as *K. oxytoca*) were screened for the presence of the *pehX* gene. As Figures 1B–D show that 12 from the 18 tested strains generated 343-bp amplicons with primer pair KO(F) and KO(R). For two strains, 0.061 and 0.067, the bands were barely visible. Thus, DNA from the 0.061 and 0.067 strains, together with those that did not generate the 343-bp fragment (0.010, 0.011, 0.013, 0.021, 0.050, 0.055, and 0.071), was checked for the presence of gene *rpoB*. Surprisingly, all tested strains were positive upon PCR with primer pairs (KP(F) and KP(R)), and 108-bp amplicons were obtained, which presumably indicated these isolates as *K. pneumoniae* (Figure 2).

### 3.3 Results of the MALDI Biotyper classification

The final part of the study includes the species classification of the tested *Klebsiella* isolates using MALDI-TOF MS Biotyper (Bruker). 17 strains were classified as *K. oxytoca*, 17 – as *K. pneumoniae* and one strain, 0.011, was identified as *Raoultella ornithinolytica*. For all strains, score values ≥2 were obtained, which proved high-quality identification (Table 3).

The *m/z* data obtained in the present study were analyzed in relation to the identified markers (Merla et al., 2019). Many peaks appeared to be common for the majority of strains tested (e.g., *m/z* at 2067, 2704, 2837, 3134, 3578, 4731, 5410, and 6254/55). As shown in Table 4, the tested strains could not be classified to determine the phylogroup. One *m/z* value, 5052, which is typical only for two phylogroups, was found for four strains tested (0.013, 0.054, 0.067, and 0.071), suggesting their affiliation with Ko1 or Ko6 (Table 4). For *K. oxytoca* 0.071 and 0.013 strains, only one specific peak was observed, whereas other strains from Table 4 shared peaks with the other isolates. For example, three other specific peaks at *m/z* 2591, 3132, and 2702 were observed for the *K. oxytoca* 0.055 strain, and they were described by Merla et al. (2019) as those associated with the Ko1, Ko4, Ko6, Ko2 (*m/z* 2591, 3132) and Ko1, Ko4, Ko6, Ko3, Ko2 (*m/z* 2702) phylogroups (Table 4).

**Table 4:** Phylogroup-specific peaks found for the selected strains from the *K. oxytoca* group.

*m/z*	Peak intensity	Strain**	Phylogroup(s) of the *K. oxytoca* complex according to the scheme proposed by Merla et al. (2019)
2591	1797	55	Ko1, Ko2, Ko4, Ko6
3132	1227, 2041, 2431, 3133	21, 55, 61, 10	
3949	1287	61	
6767	7139	21	
7109	2372, 4707	21, 61	
7898	1681	21	
4126	6456, 17575	21, 61	Ko1, Ko4, Ko6, Ko3, Ko8
5052	3543, 16219, 9128, 8341	67, 71, 13, 54	Ko1, Ko6
2702	1383, 2984, 3106	21, 55, 61	Ko1, Ko2, Ko4, Ko6, Ko3
3554*	1734, 2277, 1582	10, 61, 55	Ko1, Ko2, Ko4, Ko6
9482*	1092, 1687, 1581	55, 10, 67	Ko3, Ko6, Ko8

*Not classified as the phylogroup biomarker. **The strain numbers are simplified to make the table clearer.

## 4 Discussion

For many years, the diagnostic methods of species identification were focused on phenotypic characterization (Hansen et al., 2004). However, commercial biochemical tests for *Enterobacteriaceae* often fail to differentiate *Klebsiella* species (Chander et al., 2011; Hasan and Mohammed Bakr, 2022). The results by api 20E did not fully confirm the species affiliation indicated by the laboratory from which the strains came (Table 3). Strain 0.021 previously classified as *K. pneumoniae*, appeared to be *K. oxytoca* according to the api 20E numerical profile, whereas strain 0.034 previously designated as *K. oxytoca* was identified by api 20E as *K. pneumoniae*. However, the api 20E system also failed to identify one strain, 0.069, for which no appropriate numerical profile could be found in the Analytical Profile Index (Table 3).

In the biochemical studies, we aimed to check if the api 20E-based identification correlates with that made by using the three methods: indole test, growth determination at 10°C, and gas production from lactose at 44.5°C. As shown in Tables 2, 3, only the indole test proved to be fully effective in the differentiation between *K. oxytoca* (positive results) and *K. pneumoniae* (negative results), and its outcomes were consistent with those obtained by api 20E and MALDI Biotyper (Tables 2, 3). Such results indicate the indole assay as a reliable tool in the differentiation between *K. oxytoca* and *K. pneumoniae*. The data presented by Hansen et al. (2004) also showed this test to be effective for *K. oxytoca* identification (90–100% of the tested samples were positive for *K. oxytoca*).

The results obtained by the method of detecting gas production from lactose were not as evident as those of the indole test. This ability should be expressed by the majority of *K. pneumoniae* strains, which was proved by Hansen et al. (2004), who observed the positive results of the method for 89.3% of *K. pneumoniae* isolates. From the strains classified by api 20E (in the present study) as *K. pneumoniae*, 62.5% produced gas in EC broth and 50% in 1% lactose broth. Only 25% of *K. pneumoniae* strains showed gas production in both media. According to the technical data of the HiMedia Laboratories, the observed differences may result from the presence of the EC broth of phosphate salts controlling the pH during the fermentation process. False-negative reactions may occur due to a low pH. Obtaining more positive results in EC broth indicates that this medium is more appropriate for the detection of gas production from lactose at 44.5°C than 1% lactose broth. In the studies by Hansen et al. (2004), 3.3–6.7% of *K. oxytoca* strains produced gas from lactose. In this work, the *K. oxytoca* strains were unable to do that (Table 2), which is typical for this species. The negative results achieved for some *K. pneumoniae* strains may have resulted from the fact that they represent subspecies other than *pneumoniae*. Most *K. pneumoniae* ssp. *rhinoscleromatis* strains did not produce gas from lactose at 44.5°C, and only for 3.7–11.1% of *K. pneumoniae* spp. *ozaenae* gas was detected (Hansen et al., 2004). Interspecies differences in the ability to ferment lactose at 44.5°C among *K. pneumoniae* suggested this method as a questionable tool for *Klebsiella* species differentiation. However, this procedure is regarded as an effective method for detecting fecal coliform-positive *Klebsiella* in the environment and monitoring fecal contamination (Naemura and Seidler, 1978).

From all biochemical methods applied in the present study, a test for growing at 10°C showed the worst differentiating effect. This ability should be typical for *K. oxytoca* and not expressed by *K. pneumoniae*. However, when Hansen et al. (2004) analyzed the results obtained by one of the three laboratories, it was revealed that 57% of *K. pneumoniae* isolates were also capable of growing at 10°C. Naemura and Seidler indicated 72 h of incubation as a time adequate for noting a growth response at 10°C. In the present study, almost all strains tested, except *K. oxytoca* 0.063, grew after 72 h of incubation. In this case, the better discrimination power of the method was observed after 24 h of incubation when most of the tested *K. pneumoniae* strains did not grow, whereas most of the *K. oxytoca* isolates were capable of growing (OD_600_ > 0.02). The increase in OD values observed for 48 h-cultured and 72 h-cultured strains, independently of the species they represent, suggests that time extension is unsuitable for *Klebsiella* species differentiation.

Ambiguous results and interspecies differences observed for some biochemical reactions could result from strains’ phenotypic variations modified by environmental conditions. Thus, the standardization of biochemical tests for distinguishing *K. oxytoca* from *K. pneumoniae* is not an easy task. Moreover, using phenotypic markers for species distinguishing is not very reliable; it is time-consuming and often does not allow correcting the identification since related species frequently present similar biochemical patterns (Chander et al., 2011, Hasan and Mohammed Bakr, 2022). In contrast, PCR-based methods using different specific primers and amplification conditions have been widely suggested as reliable diagnostic tools for the correct identification of *K. pneumoniae* and *K. oxytoca* (Chander et al., 2011; Hasan and Mohammed Bakr, 2022; Kovtunovych et al., 2003). Two pairs of specific primers were proposed to be used in distinguishing these two groups of species: targeting the polygalacturonase gene and gene *rpoB*. These specific primers were also used in this study for PCR performed under the conditions described by Chander et al. with a few modifications (Table 1). For all strains biochemically identified as *K. pneumoniae,* 108-bp amplicons were obtained, which indicates the presence of the *rpoB* gene in the genome of the isolates tested. However, the *K. oxytoca* and *E. coli* reference strains also demonstrated a PCR product with 108 bp, which was only slightly visible in gel compared to those obtained for the *K. pneumoniae* isolates (Figure 1A). The *rpoB* is one of the genetic markers tested during the 16S rRNA gene sequence analysis. Recent phylogenetic studies conducted on 37 *K. pneumoniae* strains revealed that the *rrs* gene sequences coding for 16S rRNA were > 98.8% similar (Rodrigues et al., 2019). Thus, it can be seen that 16S rRNA is not always a proper target for species identification within *Enterobacteriaceae* (Cuénod et al., 2021).

The strains designed by API as *K. oxytoca* were also identified using specific primers for *pehX* gene detection. A PCR amplicon of 343 bp was obtained for twelve isolates, but for two of them (0.061 and 0.067) the product was obtained in small amounts (the slightly visible bands in Figures 1C, D). It is worth mentioning that *K. michiganensis* has been reported to be negative for *pehX* by PCR (Yang et al., 2022). Using PCR with the same set of primer pairs and similar PCR conditions, Chander et al. (2011) also did not confirm the biochemical identification of 20% of the tested *K. oxytoca* strains of human origin. The BLAST analysis indicated that *pehX* was truncated between nucleotides 1,977 and 1,983 in a few *K. oxytoca* genomes, including *K. michiganensis* strains A10 and A11, *K. oxytoca* strain 112, and *K. pasteurii* strain (Yang et al., 2022). For such strains, the detection of the *pehX* product by PCR could be hampered. The results of biochemical assays and PCR obtained for strains 0.050 and 0.034, previously identified as *K. oxytoca*, appeared to be typical for *K. pneumoniae* species. It is an unusual situation since in most cases strains previously attributed to *K. pneumoniae* appeared to be *K. oxytoca*, like in the studies of Kovtunovych et al. (2003), who developed a PCR specific for *K. oxytoca*. They succeeded in identifying six *K. oxytoca* strains previously deposited as *K. pneumoniae* in international collections.

**Figure 3:**
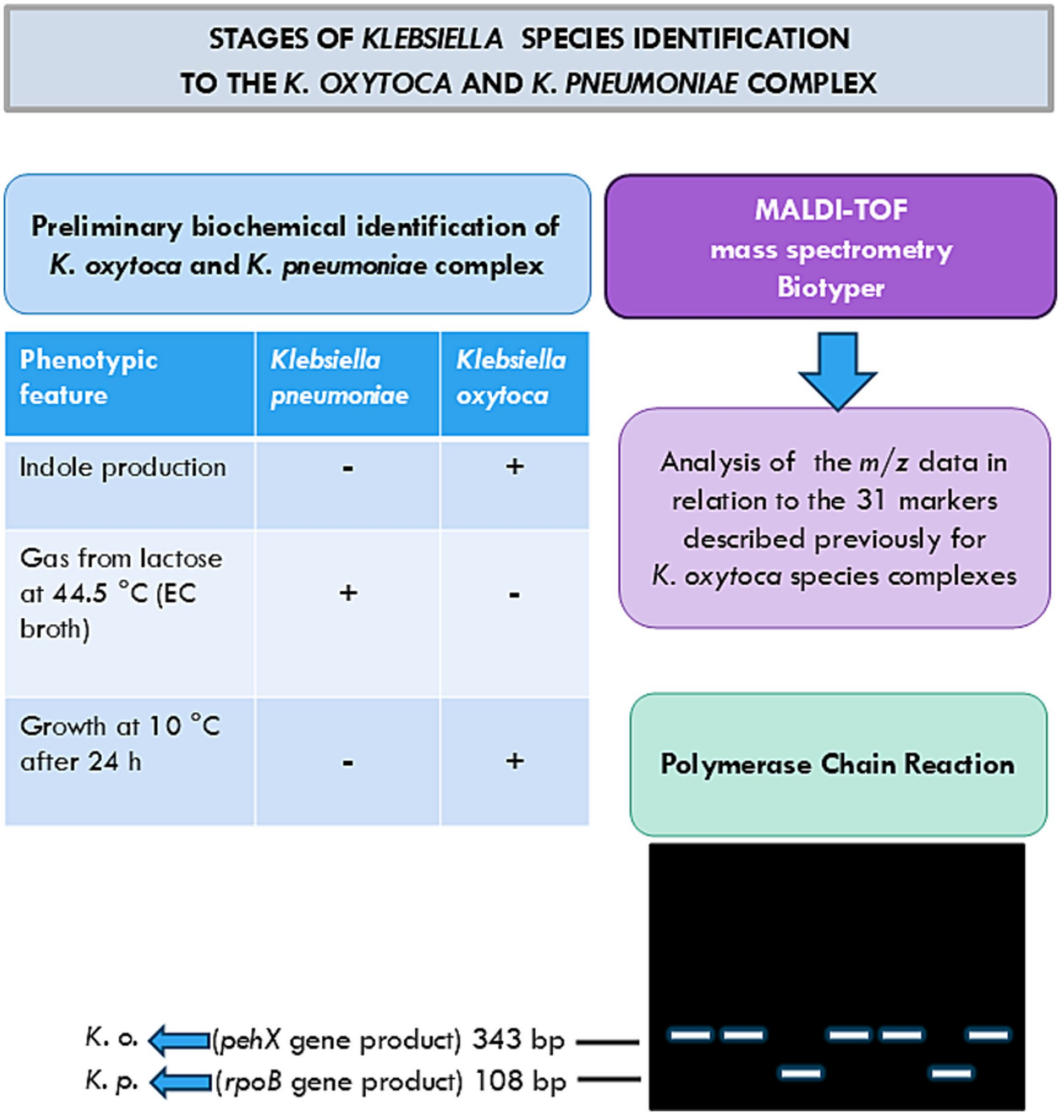
Stages of *Klebsiella* species identification by the *K. oxytoca* and *K. pneumoniae* complex used in these studies (Chander et al., 2011; Hansen et al., 2004; Kovtunovych et al., 2003; Merla et al., 2019).

The last part of the studies concerned species identification by the use of MALDI-TOF mass spectrometry analysis, the method that is increasingly applied for *Klebsiella* species identification (Figure 3) (Bridel et al., 2021; Cuénod et al., 2021; Merla et al., 2019; Rodrigues et al., 2019). The species identification by the MALDI Biotyper is consistent with the one obtained by api 20E; however, the latter appeared to have a lower ability to discriminate than the MALDI Biotyper (Tables 2, 3). Moreover, one strain, 0.069, which could not be identified by api 20E, as defined by the MALDI Biotyper as *K. pneumoniae* with a high probability of identification (Tables 2, 3).

One strain (0.011), biochemically identified as *K. oxytoca,* was designed by the MALDI Biotyper as *Raoultella ornithinolytica* with an identification rate equal to 2.27 (Table 3). Furthermore, Merla et al. identified seven strains of Ko3 and Ko8 as *Raoultella ornithinolytica*, which they explained by the lack of reference spectra of most phylogroups in the reference database. Brisse et al. (2006) noticed that the phylogenetic analysis of the *rpoB* gene sequence did not demonstrate the monophyly of *Raoultella*. Moreover, MALDI-TOF MS spectra and many biochemical features, including indole production, growth at 10°C, and gas production from lactose at 44.5°C are very similar for *K. oxytoca* and *Raoultella ornithinolytica*, which significantly hampers these species’ differentiation (Hansen et al., 2004; Merla et al., 2019).

Merla et al. (2019) also provided 31 identification biomarkers for all members of the *K. oxytoca* species complex based on a MALDI-TOF mass spectrometry analysis obtained for 30 strains from all phylogroups described so far: Ko1 (7), Ko2 (5), Ko3 (4), Ko4 (5), Ko6 (6), and Ko8 (3) (Figure 3). Analysis of the raw data obtained in the present study enabled the identification of a few m/z values assigned by Merla et al. (2019) to the *Klebsiella oxytoca* phylogroups. As Merla et al. observed, some peaks are shared by a few phylogroups; e.g., Ko4 shared most of its spectral peaks with Ko1 and Ko6, which can also be seen in Table 4 (Merla et al., 2019). To the Ko1, Ko4, Ko6, and Ko2 phylogroups, nine different m/z values were assigned: 2591, 5187, 3132, 6266, 3949, 7898, 6135, 7109, and 6767 (Merla et al., 2019). Thus, detecting these values in a strain may confirm its belonging to one of the mentioned phylogroups. For example, for *K. oxytoca* strains 0.021 and 0.061, four and three of these values were, respectively, detected (Table 4). Only for the Ko2, Ko3, and Ko8 phylogroups, specific peak combinations were proposed as biomarkers for discriminating each of these groups (Merla et al., 2019), and these peak positions could not be found in the present studies. For the *K. oxytoca* 0.013 and 0.071 strains, one specific peak was found (5052 *m/z*) reported by Merla et al. as that specific to Ko1 and Ko6 phylogroups.

The present studies indicate the MALDI-TOF mass spectrometry analysis as the most reliable method of species differentiation between *K. oxytoca* and *K. pneumoniae* groups, which also enables indicating a phylogroup within these groups. This method has been increasingly used for bacterial identification by clinical laboratories, as it is less costly than genetic identification through whole-genome sequencing, which requires DNA isolation and multi-stage bioinformatics analysis with specialized software. However, the assignment of the strains within the *K. pneumoniae* or *K. oxytoca* complex to an appropriate phylogroup is more difficult and requires reproducing similar parameters such as the standards, database, and software, since small differences in these conditions may result in slightly different *m/z* values. When *K. pneumoniae* (*n* = 10), *K.* var*iicola* (*n* = 10), *K. oxytoca* (*n* = 8), *K. michiganensis* (*n* = 12), and *K. grimontii* (*n* = 10) were analyzed on four different MALDI-TOF MS systems in various laboratories, the reproducibility of detected marker masses and the sensitivity at the species level varied depending on the system, which was explained by differences in the quality of mass spectra. Mass spectral quality is crucial in distinguishing species within the *K. oxytoca* group, as the ribosomal marker masses are found in a high mass range (Cuénod et al., 2021). MALDI Biotyper library versions are very important, as differences between versions may lead to species misidentification. For example, Ohama et al. reported the misidentification of *K. quasipneumoniae* as *K. pneumoniae* when using MALDI Biotyper version 9, whereas version 57 provided the correct identification (Ohama et al., 2022). It is worth remembering that the phylogenetic division of the *K. oxytoca* and *K. pneumoniae* complexes into phylogroups has been made quite recently, and MALDI-TOF MS analyses using more strains are desirable. The Kleborate framework is also being increasingly applied, primarily for tracking pathogens and AMR determinants (Lam et al., 2021). Using this tool solely for taxonomic assignment to species and subspecies still requires whole-genome sequencing (WGS), which is not a fully accessible method. Nevertheless, WGS data provide the information important for both methods of species identification, Kleborate and MALDI-TOF MS, and enhance the database with species-specific phylogenetic markers.

In future studies, the authors will consider sequencing the whole genomes of the tested strains or sequencing the amplicons in the search for the phosphoenolpyruvate mutase gene, *tctA* (tripartite tricarboxylate transporter gene), diguanylate phosphodiesterase gene (for the identification of *K. pneumoniae* complex species), and *bla*_OXY-(1–9)_ genes (for the identification of *K. oxytoca* complex species) (Cosic et al., 2021; Barrios-Camacho et al., 2021; Yang et al., 2022) (Supplementary Figure S2).

## Data Availability

The original contributions presented in the study are included in the article/Supplementary material, further inquiries can be directed to the corresponding author/s.
