# Supplementary material for: The results of polymerase chain reaction and MALDI-TOF mass spectrometry versus phenotypic distinction between Klebsiella pneumoniae and Klebsiella oxytoca
Source: Front Microbiol. 2025 Feb 27;16:1514643. doi: 10.3389/fmicb.2025.1514643 (PMC11903426; doi:10.3389/fmicb.2025.1514643)
Supplement: Supplementary file 1 [file Data_Sheet_1.docx]

Supplementary Material

## Supplementary Figure S1


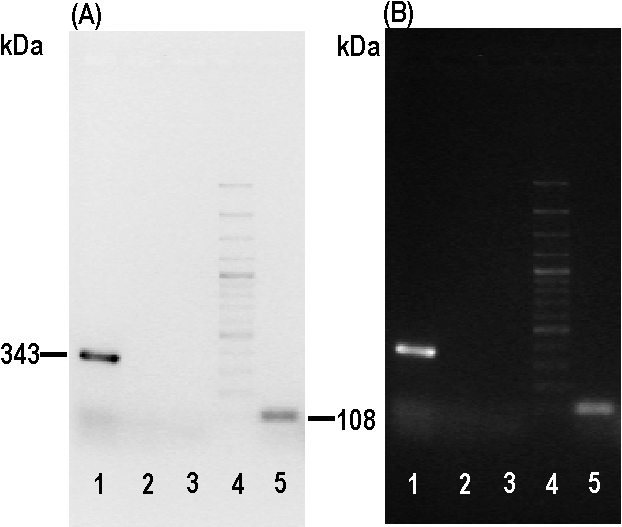


**Supplementary Figure 1.** Results of PCR amplifications of the *pehX* and *rpoB* genes obtained for the type strains: path 1, *K*. *oxytoca* ATCC 8724 (positive control of *pehX* primers; expected size of product: 343-bp) and path 5, *K*. *pneumoniae* ATCC 700603 (positive control of *rpoB* primer pairs; expected size of product: 108-bp). The order of samples 2-4 is as follows: path 2, PCR control for *pehX* primer pairs, path 3, PCR control for *rpoB* primer pairs, path 4, a 100-3000-bp ladder. The samples are presented in negative (Panel A) and in an inverted visualization option (Panel B).

Supplementary Material

## Supplementary Figure S2


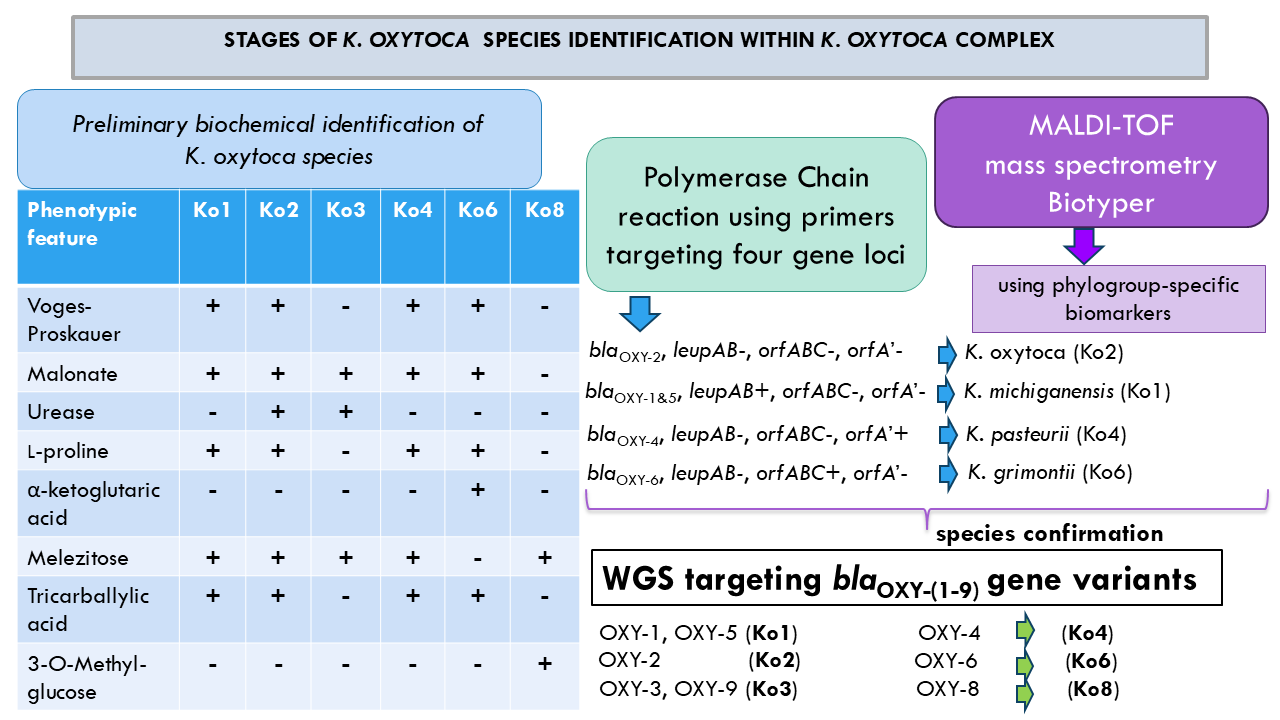


**Supplementary Figure 2.** Stages of *K. oxytoca* species identification within *K*. *oxytoca* complex (Cosic et al., 2021; Merla et al. 2019; Yang et al., 2022).
